# Impact of early antiretroviral therapy eligibility on HIV acquisition: household-level evidence from rural South Africa

**DOI:** 10.1097/QAD.0000000000001737

**Published:** 2018-03-07

**Authors:** Catherine E. Oldenburg, Jacob Bor, Guy Harling, Frank Tanser, Tinofa Mutevedzi, Maryam Shahmanesh, George R. Seage, Victor De Gruttola, Matthew J. Mimiaga, Kenneth H. Mayer, Deenan Pillay, Till Bärnighausen

**Affiliations:** a Department of Epidemiology, Harvard T.H. Chan School of Public Health, Boston, Massachusetts; b Francis I. Proctor Foundation, University of California, San Francisco, California; c Department of Global Health, Boston University School of Public Health, Boston, Massachusetts, USA; d Africa Health Research Institute, KwaZulu-Natal, South Africa; e Department of Global Health and Population, Harvard T.H. Chan School of Public Health, Boston, Massachusetts, USA; f Institute for Global Health, University College London; g Research Department of Infection and Population Health, Centre for Sexual Health, University College London, London, UK; h Faculty of Health Sciences, University of KwaZulu-Natal, Durban, South Africa; i Department of Biostatistics, Harvard T.H. Chan School of Public Health; j The Fenway Institute, Fenway Community Health, Boston, Massachusetts; k Departments of Behavioral and Social Sciences & Epidemiology, The Institute for Community Health Promotion, Brown University School of Public Health, Providence, Rhode Island; l Department of Medicine, Beth Israel Deaconess Medical Center, Boston, Massachusetts, USA; m Division of Infection and Immunity, University College London, London, UK; n Heidelberg Institute of Public Health, University of Heidelberg, Heidelberg, Germany.

**Keywords:** antiretroviral therapy, HIV incidence, regression discontinuity, South Africa, treatment as prevention

## Abstract

**Objectives:**

We investigate the effect of immediate antiretroviral therapy (ART) eligibility on HIV incidence among HIV-uninfected household members.

**Design:**

Regression discontinuity study arising from a population-based cohort.

**Methods:**

Household members of patients seeking care at the Hlabisa HIV Treatment and Care Programme in rural KwaZulu-Natal South Africa between January 2007 and August 2011 with CD4^+^ cell counts up to 350 cells/μl were eligible for inclusion if they had at least two HIV tests and were HIV-uninfected at the time the index patient linked to care (*N* = 4115). Regression discontinuity was used to assess the intention-to-treat effect of immediate versus delayed ART eligibility on HIV incidence among household members. Exploiting the CD4^+^ cell count-based threshold rule for ART initiation (CD4^+^ < 200 cells/μl until August 2011), we used Cox proportional hazards models to compare outcomes for household members of patients who presented for care with CD4^+^ cell counts just above versus just below the ART initiation threshold.

**Results:**

Characteristics of household members of index patients initiating HIV care were balanced between those with an index patient immediately eligible for ART (*N* = 2489) versus delayed for ART (*N* = 1626). There were 337 incident HIV infections among household members, corresponding to an HIV incidence of 2.4 infections per 100 person-years (95% confidence interval 2.5–3.1). Immediate eligibility for treatment reduced HIV incidence in households by 47% in our optimal estimate (hazard ratio = 0.53, 95% confidence interval 0.30–0.96), and by 32–60% in alternate specifications of the model.

**Conclusion:**

Immediate eligibility of ART led to substantial reductions in household-level HIV incidence.

## Introduction

Antiretroviral therapy (ART) is highly effective in reducing HIV transmission in serodiscordant couples [1–8]. The landmark HPTN052 trial demonstrated a 96% reduction in linked HIV transmissions in couples who immediately initiated ART compared with deferred ART initiation [9]. The causal mechanism for this dramatic reduction in HIV acquisition is primarily biological [10,11]. Early initiation of ART results in rapid and sustained viral suppression over time, whereas individuals who delay ART initiation are more likely to have a detectable viral load [12]. Although some evidence has suggested there may be changes in condom use following early initiation of ART [13], the primary protection against exposure to HIV among HIV-uninfected partners is likely via viral suppression.

Recent evidence has also documented substantial decreases in HIV transmission with the expansion of ART coverage at the community level [14,15]. This association is likely a result of both biological and social and behavioral mechanisms. Increasing ART coverage likely results not only in reduced community viral load, reducing the probability of transmission at the community level, but also affects HIV incidence through more indirect channels including increased HIV testing and counseling, changes in sexual behaviors, and changes in ART optimism that affect behaviors [14,16]. The effects of ART uptake may have spillover effects affecting HIV transmission via pathways outside of immediate sexual relationships.

Between the community and individual relationship level, there may also be unique pathways between ART uptake and HIV incidence within households [17]. At a more proximal level than the community, individuals initiating ART in households may have social influence over HIV-uninfected household members. In addition to direct biological mechanisms via cohabitating sexual partners, individuals who initiate ART may be more willing to disclose their serostatus to their families [18] and may discuss HIV prevention or elements from counseling with family members, which could result in changes in HIV acquisition in households. This could result in spillover effects including changes in sexual behaviors among household members of ART patients, such as increases in condom use or reductions in number of partners. Previous work has demonstrated a benefit of increasing coverage of ART among opposite-sex household members on HIV transmission [19]; however, the effect on all household members is unknown.

A critical issue with the identification of effects of ART in population-based surveillance cohorts is the reliance on observational data. A number of techniques meant to improve causal inference in nonrandomized studies exist, each of which contains a set of assumptions for making valid inferences [20–25]. Here, we apply a quasi-experimental approach, regression discontinuity, to estimate the causal effect of immediate versus delayed ART initiation on HIV incidence in household members.

## Methods

### Participants and procedures

Data for this analysis arose from the population-based longitudinal surveillance program conducted by the Africa Health Research Institute [26]. The surveillance program is located in a predominantly rural community of uMkhanyakude district, KwaZulu-Natal, and has been active since 2003. It includes confidential HIV testing, household demographic data, sexual history and behaviors, and relationship status. In addition to the longitudinal population-based surveillance, longitudinal data are routinely collected from the Hlabisa HIV Treatment and Care Programme, a system of public ART clinics serving the geographic area participating in the surveillance program. As the primary provider of HIV care in the area, this system captures all linkages to ART care, longitudinal CD4^+^ cell counts (measured every 6 months), and dates of ART initiation. Ethical approval for data collection, linkage, and analysis was obtained from the University of KwaZulu-Natal Biomedical Research Ethics Committee. Written informed consent was obtained from all participants. This analysis was exempted from additional ethical review by the Harvard School of Public Health Institutional Review Board due to use of anonymized secondary data.

As part of the routine demographic surveillance, information is collected about living arrangements of each participant [19]. During each surveillance round, the physical place of resident for the participant (henceforth, ‘homestead’) is recorded. A participant cannot be a member of two different homestead at the same time. Coresidents of the same homestead were defined as participants who were residents of the same homestead during a given surveillance round. Participants could move homesteads between surveillance rounds.

Participants were eligible for inclusion in this analysis if they were HIV-uninfected and a coresident of the homestead at the time the first HIV-infected partner linked to HIV care and had their first CD4^+^ cell count measured and had more than one HIV test as part of the surveillance program. Participants included in the HIV surveillance program were 15 years of age or older. Due to uncertainty in the precise timing of HIV seroconversion dates, we calculated the midpoint between the first HIV-positive test and the last HIV-negative test. We included all those individuals in the analysis who remained HIV-uninfected or whose HIV seroconversion dates were after the earliest date of linkage to HIV care for the first HIV-infected household member to link to care. We identified the date of first linkage to care as the date of the earliest CD4+ cell count recorded in the Hlabisa HIV Treatment and Care Programme. We used all earliest CD4^+^ cell counts recorded between 1 January 2007 and 1 August 2011 and between 0 and 350 cells/μl. An upper bound of 350 cells/μl was chosen, because during the study period, there were other ART eligibility thresholds at 350 cells/μl for pregnant women and tuberculosis patients. We were unable to exclude pregnant and tuberculosis patients from the dataset, because they could not be identified at the time of the earliest CD4^+^ cell count and were only identifiable for those who initiated ART. Including patients above 350 cells/μl in this circumstance would bias estimates at the 200 cell/μl threshold used in the regression discontinuity analysis. We did not place any additional restrictions on the age or sex of participants included in the analysis.

### Regression discontinuity

Regression discontinuity is a quasi-experimental study design, which can be implemented when an exposure of interest is at least in part determined by a variable measured continuously used to determine treatment or exposure status [20,24,27,28]. For example, regression discontinuity has been used to estimate the efficacy of prostate-specific antigen (PSA) screening for detection of prostate cancer [29]. Men with PSA over 4.0 ng/ml are eligible for further prostate cancer workup. The authors used PSA of more than 4.0 ng/ml as a threshold to determine whether or not a participant received additional prostate cancer workup. The authors found no decrease in prostate cancer-specific or all-cause mortality as a result of increased prostate cancer workup. Other recent examples of applications of regression discontinuity include the effect of human papillomavirus vaccination on cervical dysplasia and anal warts [30] and sexual behaviors [31], and on immediate versus delayed ART initiation on mortality [20] and retention in care [32].

We exploit the fact that immediate ART initiation upon engaging in HIV care is determined by CD4^+^ cell count. Prior to August 2011, patients in South Africa were initiated on ART if their CD4^+^ cell count fell below 200 cells/μl. The standardized monitoring schedule was CD4^+^ cell count measurement every 6 months to determine ART eligibility. If individuals presented over the 200 cells/μl threshold, they would typically not be assessed for eligibility again for 6 months, which could result in a delay in initiating ART for those who are close to the threshold. CD4^+^ cell counts are measured with some degree of error. For individuals who engaged in care with CD4^+^ cell counts of approximately 200 cells/μl, whether or not they presented just above or just below the threshold is approximately random due to the presence of measurement error [20,24].

Regression discontinuity designs are particularly useful in the setting of unmeasured confounding. Whereas most regression-based confounding adjustment methods require the strong assumption of no unmeasured confounding, regression discontinuity designs require the far weaker assumption of continuity of potential outcomes in a narrow band around the threshold that is used to assign an exposure. Particularly when the assignment variable is measured with random error, such as CD4^+^ cell count, whether or not an individual is just above or just below the threshold will be random and the continuity assumption will be met. Therefore, the distribution of measured and unmeasured confounders is expected to be similar on either side of the threshold for individuals presenting near the threshold [24]. In analyses of the effect of ART initiation on HIV incidence in household members, there may be multiple sources of unmeasured confounding, such as the household's tendency to seek health care, engagement in HIV prevention activities, or self-protective behaviors such as condom use or repeat HIV testing. We therefore chose the regression discontinuity design for the current study.

The assumption of randomness across the threshold is less likely to hold at larger distances from the threshold. This has two primary implications for the analysis and interpretation of results. First, regression discontinuity generates estimates of local effects, which are effects in the CD4^+^ cell count range close to the 200 cells/μl threshold. This has important generalizability implications, as the effect must be interpreted as the effect for patients who have an earliest CD4^+^ cell count of approximately 200 cells/μl. To estimate these local effects, regression models are estimated with separate slopes on either side of the threshold and an intercept change at the threshold. The effect estimate is the comparison of regression predictions just above versus just below the threshold (intercept shift).

Second, because regression discontinuity estimates local effects, analyses are typically presented for a range of bandwidths around the eligibility threshold that is applied to the continuous assignment variable (in this case, CD4^+^ cell count). Narrow bands represent the least biased effect estimates, because the assumption that individuals immediately above and below the threshold are similar with respect to their baseline characteristics is most likely to hold. In practice, however, there may be a limited number of individuals who are very close to the threshold. Wider bands around the threshold will improve power by including more individuals in the analysis, but will also increase potential for bias if the true relationship is nonlinear, as the local linear model will be a poorer fit to the data and lead to boundary bias at the threshold. Modeling of the assignment variable on either side of the threshold can allow for inclusion of individuals far from the threshold without substantially increasing the risk of bias if the relationship between the log-hazards and the covariates is approximately linear. Presentation of results at multiple thresholds, including narrow thresholds (with lower power and lower risk of bias), and wider thresholds (with higher power and higher risk of bias) can give additional information on the true effect size. Data-driven optimal bandwidth selectors have been derived for regression discontinuity designs using linear regression. In lieu of an optimal bandwidth, best practice is to show sensitivity to a range of bandwidth choices.

Commonly, not all patients will follow treatment assignment as determined by the assignment rule (similar to nonadherence in a randomized controlled trial). Indeed, patients may have been started on therapy with a high CD4^+^ cell count due to Stage IV HIV illness during this period of study. In this case, the intention-to-treat (ITT) effect in regression discontinuity is estimated in a regression model with a term for whether the individual was above or below the threshold and terms for the slope of the assignment variable above and below the threshold. The ITT analysis generates the effect of presenting just below the threshold, analogous to a randomized controlled trial in which the ITT analysis generates the effect of the random assignment, regardless of whether or not the patients actually adhered to their randomized treatment.

### Statistical analysis

All analyses accounted for multiple individuals within a homestead by clustering standard errors at the homestead level. We estimated the ITT effect by fitting a Cox proportional hazards model to the value of the first CD4^+^ cell count, allowing the hazard to shift at the threshold, and allowing the slope above and below the threshold to differ. Analyses were conducted in a range of bandwidths around the assignment variable as well as the optimal bandwidth determined by the Imbens–Kalyanaraman algorithm using a linear probability model [33]. This algorithm estimates the optimal bandwidth in the trade-off between statistical power and bias. We assessed robustness to modeling the relationship with the assignment variable as a quadratic and as a restricted cubic spline with knots at 100 cells/μl on either side of the threshold, allowing for nonlinear relationships between CD4^+^ cell count and the log-hazards. The restricted cubic splines relax the linearity assumption and provide information on whether our assumption of linearity in the primary models led to bias. We ran an additional sensitivity analysis including baseline covariates. If, as expected, baseline covariates are balanced above and below the threshold, there should be no change in point estimates with the inclusion of additional baseline covariates [34]. Variables included in sensitivity analyses included the age, educational status, and sex of the respondent and the household member linking to care as well as an index of the household's wealth. We used multiple specifications of the hazard function, including both exponential and Weibull distributions. We used instrumental variable methods [23] to estimate the effect of immediate initiation of ART on household HIV incidence among individuals who took treatment, because they were below the threshold (see Appendix for details). Analyses were run in Stata 14.1 (StataCorp, College Station, Texas, USA).

## Results

A total of 4115 individuals were HIV-uninfected at the time the first HIV-infected individual in their household linked to care with a CD4^+^ cell count between 0 and 350 cells/μl. Of these, there were 2490 HIV-uninfected household members among HIV-infected individuals who linked to care below 200 cells/μl (and thus eligible for ART) and 1626 above 200 cells/μl (and thus ineligible). Baseline characteristics were well balanced between those who started above and below 200 cells/μl (Table 1). Balance tests indicated no difference in baseline characteristics at the threshold.

**Fig. 1:**
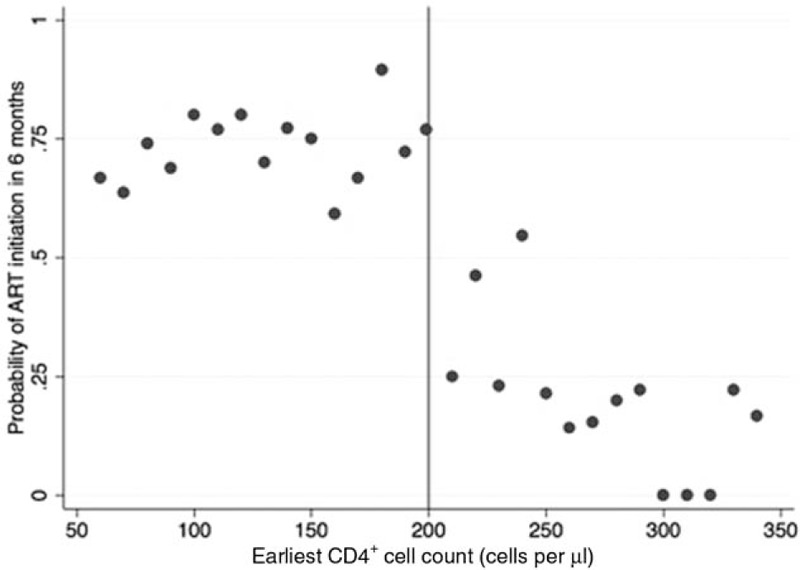
Probability of antiretroviral therapy initiation by baseline CD4^+^ cell count.

**Fig. 2:**
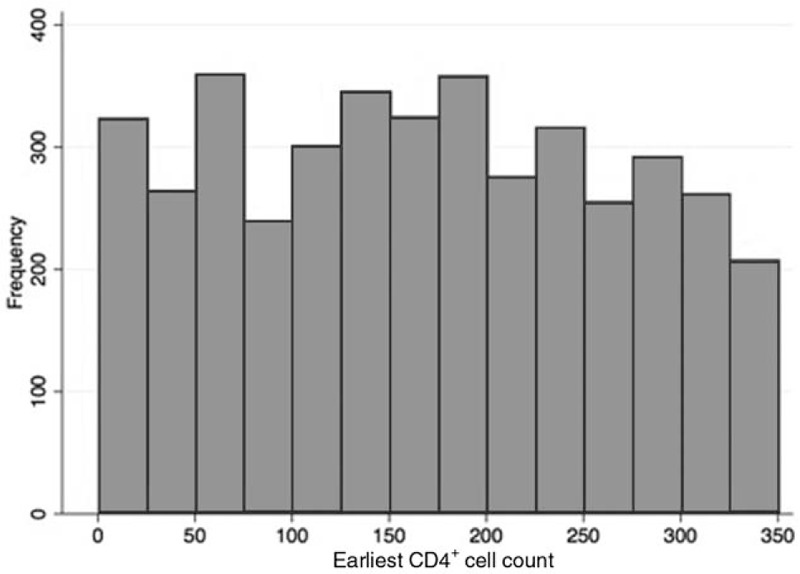
Regression discontinuity validity test – histogram displaying distribution of baseline CD4^+^ cell counts.

The Imbens–Kalyanaraman algorithm determined that the optimal bandwidth was 95 cells/μl above and below the threshold. At CD4^+^ cell count bandwidths of 0–350, 50–350, 105–295 (the optimal bandwidth), 150–250, and 175–225 cells/μl, a total of 4115, 3531, 2356, 1268, and 615 HIV-uninfected individuals were included in the analyses, respectively. The probability of ART initiation within 6 months of the HIV-infected household member by first CD4^+^ cell count at the clinic is displayed in Fig. 1. The probability of initiation of ART within 6 months was highest among individuals who presented below 200 cells/μl, and there was a strong discontinuity at the threshold. A histogram of baseline CD4^+^ cell counts (Fig. 2) demonstrated no bunching at the threshold, indicating that manipulation of CD4^+^ cell counts, which could bias results, is unlikely.

The probability of antiretroviral therapy initiation within 6 months of initiating HIV care was calculated as the number of individuals who initiated antiretroviral therapy within 6 months of the date of their earliest CD4^+^ cell count divided by the total number of individuals who had a first CD4^+^ cell count date. Probabilities were calculated by 10-cells/μl bins of earliest CD4^+^ cell counts (the first CD4^+^ cell count the individual had after initiating HIV care).

The distribution of frequency of baseline CD4^+^ cell counts of the first member of the household to link to HIV care. This histogram demonstrates no evidence of bunching at the threshold (200 cells/μl), indicating no evidence of manipulation of baseline CD4^+^ cell counts. Evidence of bunching could be seen if there were substantially more patients in the bin immediately above (or the bin immediately below) the threshold compared with the other side. Manipulation of baseline CD4^+^ cell counts, which could occur for example if clinicians reported a CD4^+^ cell count below 200 cells/μl when the true CD4^+^ cell count was above 200 cells/μl so the patient could access antiretroviral therapy, would result in biased estimates of the effect of antiretroviral therapy eligibility on outcomes. Manipulation could occur, for example, if clinicians reported a CD4^+^ cell count below 200 cells/μl when the true CD4+ cell count was above 200 cells/μl, so the patient could access antiretroviral therapy.

**Fig. 3:**
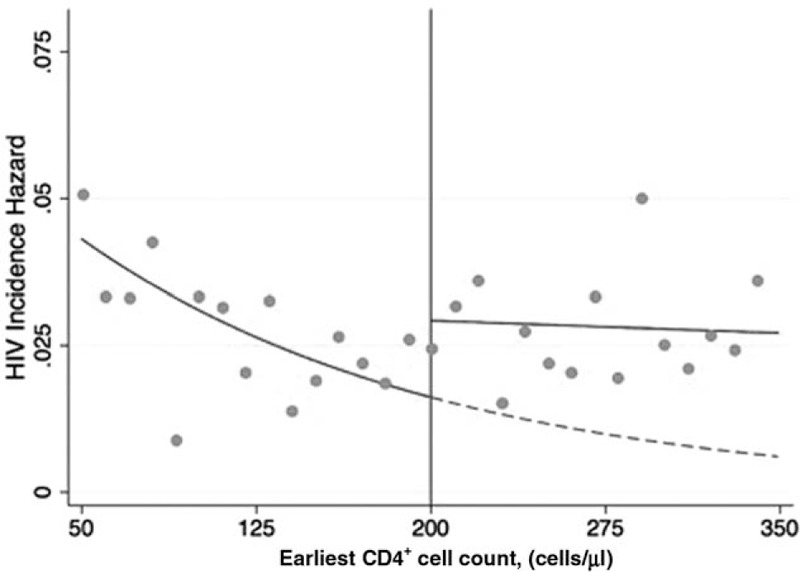
HIV incidence by baseline CD4^+^ cell count.

There were 337 HIV seroconversions among 13 785 person-years at risk, with an overall incidence rate of 2.4 seroconversions per 100 person-years [95% confidence interval (CI) 2.2–2.7]. The discontinuity in HIV incidence at the threshold by baseline CD4^+^ cell count of the first HIV-infected household member to link to care is shown in Fig. 3. In the optimal bandwidth (105–295 cells/μl), immediate initiation of ART reduced HIV incidence by 47% (hazard ratio 0.53, 95% CI 0.30–0.96), Table 2. Models at wider bandwidths that included more flexible modeling of the functional form of CD4^+^ cell count were consistent with a 50% reduction in HIV incidence, similar to effect estimates at the narrower bandwidths. Sensitivity analyses modeling CD4^+^ cell count with restricted cubic splines and squared terms allow for flexible modeling of the relationship between CD4^+^ cell count and HIV incidence. These models may reduce bias in effect estimates at the widest bandwidth, in which individuals are included further from the threshold, by improving model fit. In the widest bandwidth (0–350 cells/μl), which includes the most information but is the most sensitive to violations of the assumption of linearity, the hazard ratio with a linear functional form of CD4^+^ cell count was 0.68 (95% CI 0.46–1.02), which decreased to 0.48 (95% CI 0.26–0.88) with a restricted cubic spline at 100 cells/μl above and below the threshold and 0.45 (95% CI 0.24–0.85) with a squared term for CD4^+^ cell count. At the narrowest bandwidth (175–225), which has the least power but is the least vulnerable to misspecification, the hazard ratio was 0.40 (95% CI 0.14–1.13). These results were robust to alternative specifications of the hazard function and inclusion of baseline covariates in the model (Supplemental Tables S1 and S2). HIV incidence among household members who initiated treatment, because they were below the threshold, had a 93% reduction in HIV incidence compared with those who did not initiate treatment, because they were above the threshold (hazard ratio 0.07, 95% CI 0.01–0.52; Appendix).

HIV incidence in HIV-uninfected household members by the baseline CD4^+^ cell count of the first HIV-infected member of their household to link to HIV care. Orange dots indicate the raw HIV incidence for each 10 cell/μl bin. Green lines indicate fitted regression lines estimating the incidence of HIV as a function of earliest CD4^+^ cell count above and below the threshold (red line). The dotted green line is the projection for the curve below the threshold, which is the estimate of what HIV incidence for individuals above the threshold (who are not eligible for immediate antiretroviral therapy initiation) would have been, if they had actually been eligible for antiretroviral therapy immediately. The discontinuity at the threshold is the estimate of the effect of antiretroviral therapy eligibility on incidence.

## Discussion

When a person living with HIV was immediately eligible for ART, HIV incidence was reduced among HIV-uninfected household members compared to delayed ART eligibility. HIV incidence was reduced by approximately 50%. Although there was some variation with different model specifications suggesting that a range of reduction in HIV incidence could be possible, alternate specifications of the model indicate that the true effect size is likely closer to 50%. We included all individuals in the household in this analysis, regardless of whether individuals were in a sexual partnership. This study therefore suggests that there are benefits to entire households when HIV-infected household members are immediately eligible for ART. These benefits extend beyond the well documented benefits in couples [1,35,36].

As expected, the effect estimates in this study fell between those from the HPTN052 randomized controlled trial among serodiscordant couples [9] and community-level effects of ART on HIV incidence [14]. Similar to the prior community-level estimates, the effect we quantified in this study includes both biological reduction in transmission risk among sex partners to the person on ART as well as potential changes in behavior that may affect HIV acquisition among HIV-uninfected household members. The latter may include increased condom use and reduced sexual awareness and risk protection. We expected the effect measured among household members to be smaller than the effect among sero-discordant couples, because the biological efficacy of ART is nearly perfect, effectively blocking transmission among partners, while any behavioral spillover effects associated with ART use are unlikely to completely eliminate HIV acquisition risk. By contrast, we expected the effect among household members to be larger than the community-level effects of ART, because both the biological and behavioral pathways from ART utilization to HIV incidence are more direct and less diluted among household members compared to community members.

To date, the vast majority of studies assessing HIV infections within households or families have focused on HIV transmission within couples or mother to child transmission. In both scenarios, ART has been shown to be highly efficacious in the prevention of HIV infection [35,37,38]. Evidence from the United States demonstrated substantial clustering of HIV within households of HIV-infected or high-risk women [17], with household infection more common among siblings than among intimate partners or children. Residents of the same physical spaces likely share common characteristics, including socioeconomic, education, behavioral, and community characteristics that may influence HIV risk. Within households, individuals who immediately initiate ART after an initial clinic visit may more frequently disclose their status to household members, which could lead to behavioral changes among household members. HIV prevention messages from counseling in the clinic may be more likely to reach household members of those who immediately uptake ART, which could result in decreases in household HIV acquistion. In addition, immediate ART initiation will lead to earlier biological effects and – because people on ART visit clinics more frequently than people enrolled in pre-ART – stronger behavioral effects induced by exposure to HIV-related care and counseling.

The current analysis has several important limitations. Because not everyone in the surveillance system participated in HIV testing every year, the decision to participate in testing may be affected by the exposure (immediate ART initiation by the HIV-infected household member). However, data arose from two separate systems (the public-sector ART clinic data and the HIV surveillance system), and these results are unlikely due to differential nonparticipation. Without additional untestable assumptions [20,39], the results of this analysis may only be generalizable to individuals who present close to the ART initiation threshold. To generalize the results beyond the group, additional assumptions related to the functional form of how the unobserved potential outcome changes with the assignment variable over the distribution of CD4^+^ cell counts are required [39]. Because these assumptions are untestable the local effect that we have estimated may not be generalizable to a global effect across the entire range of HIV-infected individuals who visit clinics. However, this limitation concerns only external validity and the use of regression discontinuity represents a significant strength regarding internal validity, as it does not rely on the assumption of no unmeasured confounding. Furthermore, a strength of this analysis is the ability to link clinic-based data to household data, including HIV surveillance in household members, allowing for estimation of effects within households without relying on self-report from individuals linked to HIV care.

We found a substantial reduction in HIV incidence in households with immediate eligibility for ART. The results of this study provide further evidence of the importance of immediate initiation of ART to reduce HIV transmission. Furthermore, our results indicate that ART utilization induces behavior changes within households that reduce HIV incidence over and above the reductions due to the biological ART effect. Thus, ART initiation likely has benefits to members of the social network extending beyond sexual partners. Taken together, our results support policies that eliminate ART eligibility thresholds and interventions to rapidly increase ART coverage through expansions of HIV treatment programs.

## Acknowledgements

Author contributions: C.E.O. designed the study, conducted analyses, and drafted the report. J.B. designed the study, conducted analyses, and drafted the report. G.H. interpreted results and drafted the report. F.T. supervised data collection, designed the study, interpreted results, and critically reviewed the article. T.M. supervised data collection, designed the study, interpreted results, and critically reviewed the article. M.S. supervised data collection, designed the study, interpreted results, and critically reviewed the article. G.R.S. designed the study, interpreted results, and critically reviewed the article. V.D.G. designed the study, interpreted results, and critically reviewed the article. M.J.M. designed the study, interpreted results, and critically reviewed the article. K.H.M. designed the study, interpreted results, and critically reviewed the article. D.P. supervised data collection, designed the study, interpreted results, and critically reviewed the article. T.B. supervised data collection, designed the study, conducted analyses, and drafted the report.

The Hlabisa HIV Treatment and Care Programme was funded by the United States Agency for International Development and the President's Emergency Plan (grant 674-A-00-08-001-00). This work was partially supported by the National Institutes of Health (grants T32-DA013911 and R25-MH083620 to C.E.O., grant R37-AI51164 to V.D.G., grant R01-HD084233 to T.B., and grants R01-AI124389 and R01-HD084233 to T.B. and F.T.). T.B. was funded by the Alexander von Humboldt Foundation through the Alexander von Humboldt Professorship endowed by the German Federal Ministry of Education and Research. The Africa Health Research Institute, University of KwaZulu-Natal, South Africa receives core funding from the Wellcome Trust (grant 082384/Z/07/Z).

### Conflicts of interest

There are no conflicts of interest.

## Supplementary Material

Supplemental Digital Content

## Figures and Tables

**Table 1:** Baseline characteristics of study sample by household member antiretroviral therapy eligibility (*N* = 4115).

	Below threshold, *N* = 2489	Above threshold, *N* = 1626	Balance test *P* value^*^
Age when first household member linked to care, years, median (IQR)	20 (16–48)	20 (16–47)	0.15
Female sex	1529 (61.4%)	1015 (62.4%)	0.70
Number of HIV-uninfected individuals in household, median (IQR)	3 (2–4)	3 (2–4)	0.58
Highest education attainment			
Less than 7 years	1026 (41.2%)	682 (41.9%)	0.38
7–12 years	1394 (56.0%)	891 (54.8%)	
More than 12 years	63 (2.5%)	51 (3.1%)	
Knows HIV status	481 (19.3%)	295 (18.1%)	0.77
Household location			
Urban	42 (1.7%)	42 (2.6%)	0.78
Peri-urban	904 (36.3%)	536 (33.0%)	
Rural	1544 (62.0%)	1048 (64.5%)	
Household distance to clinic, km (median, IQR)	2.7 (1.5–3.9)	2.7 (1.5–4.1)	0.28
Household wealth (quintile)			
Lowest quintile	420 (16.7%)	260 (16.0%)	0.31
Second lowest	481 (19.3%)	333 (20.5%)	
Middle	585 (23.5%)	375 (23.1%)	
Second highest	496 (19.9%)	356 (21.9%)	
Highest	419 (16.8%)	233 (14.3%)	
Missing	89 (3.6%)	69 (4.2%)	

IQR, interquartile range.

^*^Regression discontinuity model using each baseline covariate as the outcome model to test if there is a discontinuity at the CD4^+^ cell count thresholds. A linear regression model was used for continuous variables, logistic regression for dichotomous variables, and ordinal logistic regression for ordinal variables.

**Table 2:** Regression discontinuity intention-to-treat effects of antiretroviral therapy on household HIV incidence.

Range	*N*	Cox	Cox, quadratic	Cox, restricted cubic splines
0–350				
Immediate ART	4115	**0.68 (0.46–1.02)**	**0.45 (0.24–0.85)**	**0.48 (0.26–0.88)**
Slope above^a^		0.999 (0.996–1.003)	0.993 (0.979–1.007)	0.996 (0.987–1.006)
Slope below^b^		0.997 (0.993–1.002)	0.997 (0.980–1.014)	1.006 (0.994–1.018)
50–350				
Immediate ART	3531	**0.55 (0.35–0.86)**	**0.55 (0.28–1.08)**	**0.57 (0.31–1.05)**
Slope above^a^		0.999 (0.996–1.003)	0.993 (0.979–1.007)	0.996 (0.987–1.006)
Slope below^b^		0.994 (0.989–0.999)	1.007 (0.986–1.028)	0.990 (0.971–1.010)
105–295^c^				
Immediate ART	2356	**0.53 (0.30–0.96)**	**0.46 (0.21–1.00)**	**0.48 (0.24–0.98)**
Slope above^a^		0.998 (0.991–1.006)	0.973 (0.949–0.997)	0.983 (0.968–0.999)
Slope below^b^		0.995 (0.984–1.005)	1.035 (0.996–1.076)	0.941 (0.854–1.037)
150–250				
Immediate ART	1268	**0.47 (0.23–0.98)**		
Slope above^a^		0.986 (0.969–1.002)		
Slope below^b^		1.014 (0.988–1.040)		
175–225				
Immediate ART	615	**0.40 (0.14–1.13)**		
Slope above^a^		0.980 (0.938–1.023)		
Slope below^b^		1.001 (0.938–1.080)		

ART, antiretroviral therapy. The numbers shown in bold font are the main effect size estimates.

^a^Difference in slope of CD4^+^ cell count above the 200 cell/μl threshold.

^b^Difference in slope of CD4^+^ cell count bellow the 200 cell/μl threshold.

^c^Optimal bandwidth determined by the Imbens–Kalyanaraman algorithm [33].
