# Supplementary material for: Impact of early antiretroviral therapy eligibility on HIV acquisition: household-level evidence from rural South Africa
Source: AIDS. 2018 Mar 7;32(5):635–43. doi: 10.1097/QAD.0000000000001737 (PMC5832606; doi:10.1097/QAD.0000000000001737)
Supplement: Supplemental Digital Content [file aids-32-635-s001.docx]

**Table S1.** Alternate specifications of primary models with exponential and Weibull distributions.

| **Range** | **Exponential** | **Weibull** |
| --- | --- | --- |
| *0-350*  Immediate ART  Slope above^1^  Slope below^2^ | **0.68 (0.46 to 1.02)**  1.000 (0.996 to 1.003)  0.997 (0.993 to 1.002) | **0.68 (0.46 to 1.01)**  0.999 (0.996 to 1.003)  0.998 (0.993 to 1.002) |
| *50-350*  Immediate ART  Slope above^1^  Slope below^2^ | **0.55 (0.35 to 0.87)**  0.997 (0.990 to 1.005)  0.996 (0.986 to 1.007) | **0.55 (0.35 to 0.86)**  0.999 (0.996 to 1.003)  0.994 (0.989 to 0.999) |
| *105-295***  Immediate ART  Slope above^1^  Slope below^2^ | **0.52 (0.29 to 0.93)**  1.000 (0.994 to 1.008)  0.994 (0.984 to 1.003) | **0.52 (0.29 to 0.93)**  0.998 (0.990 to 1.005)  0.996 (0.986 to 1.006) |
| *150-250*  Immediate ART  Slope above^1^  Slope below^2^ | **0.47 (0.23 to 0.98)**  0.986 (0.969 to 1.002)  1.014 (0.988 to 1.040) | **0.47 (0.23 to 0.98)**  0.986 (0.969 to 1.002)  1.014 (0.988 to 1.040) |
| *175-225*  Immediate ART  Slope above^1^  Slope below^2^ | **0.39 (0.14 to 1.13)**  0.979 (0.937 to 1.023)  1.008 (0.939 to 1.081) | **0.40 (0.14 to 1.13)**  0.979 (0.938 to 1.023)  1.007 (0.939 to 1.081) |

^**^Optimal bandwidth per Imbens-Kalyanaraman algorithm; ^1^Difference in slope of CD4 count above the 200 cell/μl threshold; ^2^Difference in slope of CD4 count bellow the 200 cell/μl threshold

**Table S2.** Sensitivity analyses for the intention-to-treat effect of ART eligibility on household HIV incidence

| **Range** | **N** | **Spline** | **Adjustment for Age and Sex** | **Spline + Adjustment for Age and Sex** |
| --- | --- | --- | --- | --- |
| *0-350*  Immediate ART  Slope above  Slope below | 4,115 | **0.48 (0.26 to 0.88)**  0.996 (0.987 to 1.006)  1.006 (0.994 to 1.018) | **0.63 (0.42 to 0.95)**  0.999 (0.995 to 1.003)  0.997 (0.993 to 1.002) | **0.48 (0.27 to 0.88)**  0.995 (0.986 to 1.005)  1.004 (0.992 to 1.016) |
| *50-350*  Immediate ART  Slope above  Slope below | 3,531 | **0.57 (0.31 to 1.05)**  0.996 (0.987 to 1.006)  0.990 (0.971 to 1.010) | **0.54 (0.34 to 0.85)**  0.999 (0.995 to 1.003)  0.995 (0.990 to 1.000) | **0.56 (0.30 to 1.02)**  0.995 (0.986 to 1.005)  0.991 (0.972 to 1.010) |
| *105-295*  Immediate ART  Slope above  Slope below | 2,356 | **0.48 (0.24 to 0.98)**  0.983 (0.968 to 0.999)  0.941 (0.854 to 1.037) | **0.51 (0.29 to 0.92)**  0.997 (0.99 to 1.005)  0.993 (0.986 to 1.001) | **0.47 (0.23 to 0.96)**  0.985 (0.970 to 1.001)  0.953 (0.866 to 1.049) |
| *150-250*  Immediate ART  Slope above  Slope below | 1,268 |  | **0.46 (0.22 to 0.96)**  0.986 (0.970 to 1.002)  1.013 (0.987 to 1.039) |  |
| *175-225*  Immediate ART  Slope above  Slope below | 615 |  | **0.35 (0.12 to 1.01)**  0.982 (0.940 to 1.025)  0.995 (0.925 to 1.071) |  |

**Appendix 1: Complier average causal effects**

In addition to the ITT, the effect of therapy itself on those who take up treatment because of the threshold can be estimated. This is analogous to the effect in a randomized controlled trial of the effect of treatment itself on outcomes. The complier average causal effect (CACE) is the ITT scaled by the difference in probability of treatment at the threshold, which is equivalent to the instrumental variable estimator.[1] As with other instrumental variable frameworks[2], the CACE requires the assumptions of 1) monotonicity, which is the assumption that there are no “defiers”, that is, no patients would have initiated ART if they were ineligible and refuse treatment if they were eligible, and 2) the exclusion restriction, which states that the instrument (presenting just below the threshold) only affects HIV incidence in households through initiation of ART. As in instrumental variable approaches, if the ITT is null, the CACE will be null. If this ITT is not null, the CACE is typically further from the null than the ITT, as it is directly estimating the effect of treatment among the compliers.

Instrumental variable methods, which are used to estimate the CACE, are relatively new in application to censored survival outcomes.[3] Recently, methods have been developed for instrumental variable estimation using an Aalen additive hazards model.[3] For binary exogenous variables (i.e., presenting below the threshold), the control function approach has been shown to be appropriate.[3,4] This method involves estimating deviance residuals from a first-stage regression model and including the residuals in a second stage model. Furthermore, work has demonstrated that when the cumulative incidence of disease over the follow-up period is rare, the control function approach may be used in a Cox proportional hazards model.[3] We therefore used two approaches to estimate the CACE for the effect of immediate versus delayed ART initiation in the first individual in the household to link to HIV care and HIV incidence in households.

To estimate the CACE, we fit a first-stage logistic regression model with terms for presenting immediately above or below the threshold and the gap above and below the threshold (to allow for differential slopes above and below the threshold). Residuals were estimated from this model, which were then included in the second-stage model. The second-stage model was an Aalen additive hazards model with terms for initiating ART within 6 months, the gap above and below the threshold, and a term for residuals estimated in the first stage model. We then repeated the procedure with a Cox proportional hazards model. Because the inclusion of the residual term in the second stage model does not account for uncertainty inherent in estimating the residuals in the first stage, all inferences were based on 5,000 nonparametric bootstraps. Analyses also accounted for clustering within physical residencies (homesteads).

In a Cox proportional hazards model, the CACE of the first individual in the household to link to care to take up ART because they presented for care below the CD4 count threshold indicated more than 90% reduction in household HIV acquisition (Table 2). For the widest bandwidth, the hazard ratio was 0.07 (95% CI 0.01 to 0.52), which decreased to 0.02 (95% CI 0.0002 to 1.56) at the narrowest bandwidth. The Aalen model, in the widest bandwidth, indicated 7.5 fewer cases of HIV per year of follow-up among 100 people who initiated ART because they were below the CD4 count threshold compared to if those 100 people had not initiated ART because they were above the threshold.

| **Bandwidth** | **Cox Model** | **Aalen Model** |
| --- | --- | --- |
| 50-350 | 0.07 (0.01 to 0.52) | -0.075 (-0.13 to -0.02) |
| 100-300 | 0.06 (0.004 to 0.80) | -0.054 (-0.16 to 0.086) |
| 150-250 | 0.02 (0.0002 to 1.56) | -0.035 (-0.31 to 0.41) |

In addition to estimation of the ITT, regression discontinuity designs allow for estimation of the complier average causal effect (CACE), which is the effect of initiating ART because an individual of the threshold rule. This effect is limited in its interpretation to individuals who comply with the treatment; that is, individuals who always take treatment if they link to care below the threshold and individuals who never take treatment if they link to care above the threshold. As expected, in this study the CACE was substantially larger than the ITT. Whereas the ITT is considered the parameter of interest from a policy perspective, the CACE is likely of additional interest for clinicians. In the population of compliers, the results of this study suggest a dramatic reduction in HIV acquisition in households when the earliest member of the household to link to HIV care does so immediately below the threshold for initiating ART.

**References**

1. Bor J, Moscoe E, Mutevedzi P, Newell M-L, Barnighausen T. Regression Discontinuity Designs in Epidemiology. Epidemiology. 2014;25: 729–737. doi:10.1097/EDE.0000000000000138

2. Hernan MA, Robins JM. Instruments for Causal Inference: An Epidemiologist's Dream? Epidemiology. 2006;17: 360–372. doi:10.1097/01.ede.0000222409.00878.37

3. Tchetgen Tchetgen EJ, Walter S, Vansteelandt S, Martinussen T, Glymour M. Instrumental Variable Estimation in a Survival Context. Epidemiology. 2015;26: 402–410. doi:10.1097/EDE.0000000000000262

4. Tchetgen Tchetgen E. A note on the control function approach with an instrumental variable and a binary outcome. Epidemiologic Methods. 2014;3: 107–112. doi:10.1515/em-2014-0009
